# Pilot study: Is a long‐term follow‐up service beneficial for patients undergoing revision hip replacement surgery?

**DOI:** 10.1002/msc.1521

**Published:** 2020-10-21

**Authors:** Lindsay K. Smith, Emma Turner, Erik Lenguerrand, Jane Powell, Shea Palmer

**Affiliations:** ^1^ Faculty of Health and Applied Sciences University of the West of England Bristol UK; ^2^ Musculoskeletal Research Unit University of Bristol Southmead Hospital Bristol UK; ^3^ Sirona Care and Health CIC Bristol UK

**Keywords:** arthroplasty, follow‐up, guidelines, replacement, surveillance, total hip

## Abstract

**Purpose:**

Total hip arthroplasty (THA) is highly successful but some patients will require later revision surgery. This pilot study evaluates the effects of long‐term follow‐up for patients undergoing revision hip replacement.

**Methods:**

Consecutive patients undergoing aseptic revision of THA were recruited from a large orthopaedic unit to a single centre, observational study. Primary outcomes were changes in patient‐reported scores from pre‐revision to 12 months post‐surgery. Secondary outcomes were costs during hospital stay up to 6 months post‐revision. Participants were retrospectively allocated to two groups—those with regular orthopaedic review prior to revision (Planned revision) or those without (Unplanned revision).

**Results:**

52 patients were recruited, 7 were unrevised, one incomplete baseline questionnaires. There were 25 planned and 19 unplanned revisions with no significant differences between groups at baseline. At 12 months, 34 complete data sets were available for analysis, 17 in each group. Change scores were analysed with Mann–Whitney *U* test; none reached statistical significance. There was a significant difference for length of stay: Planned group 5 days (2–22), Unplanned 11 days (3–86) (Mann–Whitney *U* test, *p* = 0.023). No significant differences found for theatre time or component costs. Resource costs post‐revision surgery are presented.

**Conclusion:**

This pilot study indicates that some change in methods would be required for future work. The results show that there may be some financial benefit from providing long‐term follow‐up of THA but a larger study is needed to explore these findings and to discuss the impact on recommended guidelines.

## 1 INTRODUCTION

It is known that total hip arthroplasty (THA) is a successful procedure for end‐stage arthritis of the hip through relief of pain and restoration of movement and that, once in place, the joint replacement may be effective for many years (Patel, Pavlou, Mujica‐Mota, & Toms, 2015). The size of the affected population is considerable—in England, Wales, Northern Ireland and the Isle of Man, there are approximately 90,000 primary THA completed each year (National Joint Registry, 2019). Recent evidence from Australia, based on registry data from 2003 to 2013, predicts a rise of 208% on 2013 procedure numbers by 2030 (Ackerman et al., 2019). Up to 10% of these replacements will require revision during the lifetime of the recipient (National Joint Registry, 2019). Early revision, in the first 5 years, is often due to a symptomatic condition such as infection or dislocation but revision beyond the early period is more likely to be attributable to other causes such as aseptic loosening which may be asymptomatic and continues to be the most common indication for revision of a hip replacement (National Joint Registry, 2019).

Traditional guidelines, produced by specialist orthopaedic societies, recommend mid to long‐term follow‐up of this population to provide ongoing care. ‘Long‐term’ is used in reference to the period beyond 10 years (mid‐term beyond 5 years) when an assessment of joint construct and symptoms may identify any damaging changes, especially asymptomatic ones, and evaluate the need for revision (British Hip Society, British Orthopaedic Association, & Royal College of Surgeons of England, 2019). Planning for revision can potentially improve the experience and outcomes through damage limitation and pre‐operative preparation. Currently, the requirement for long‐term follow‐up is questioned in the United Kingdom and elsewhere due to changes in materials and construct design, the use of components with an Orthopaedic Date Evaluation Panel rating, patient expectations and the economic constraints on many health services (Cassidy, O Heireamhoin, & Beverland, 2019; Hacking, Weinrauch, Whitehouse, Crawford, & Donnelly, 2010; Lovelock et al., 2018).

A survey of practice across the United Kingdom in 2013 showed that only 43% of the sample of orthopaedic units included were offering any long‐term follow‐up, beyond 10 years (Smith, 2014). Many of these had changed practice because of constraints on service delivery rather than because they had clinical evidence to support the disinvestment. In a systematic literature review for evidence of the effectiveness of long‐term follow‐up after THA, there were no quantitative evaluations of such services amongst the 114 studies included (Smith, Dures, & Beswick, 2019). In the absence of this information, the research question for this pilot study was: Is there evidence to suggest a beneficial effect of follow‐up services for patients undergoing revision hip replacement? The objectives were to test the logistics of the proposed methods, and to gather information on patient reported outcomes and associated costs.

## 2 METHODS

### 2.1 Study design and participants

The detailed protocol and study design have previously been published (Smith, Lenguerrand, Blom, Powell, & Palmer, 2017). This was a pilot observational study, conducted as a preliminary investigation of the proposed methods, which took place in a single, large orthopaedic unit in the United Kingdom. The participants were all those undergoing aseptic revision of THA and were recruited consecutively over a 12‐month period, with final data collected 12 months after revision surgery. Inclusion criteria were adults over 18 years, ability to complete a postal English language questionnaire and more than 5 years between primary and revision arthroplasty.

### 2.2 Group allocation

**FIGURE 1:**
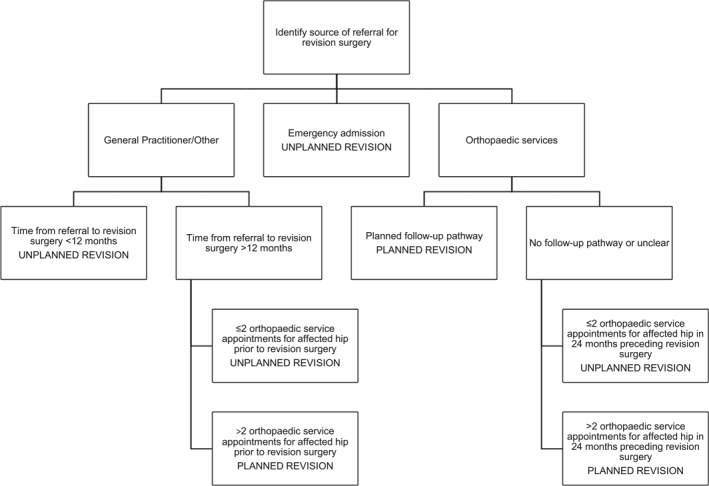
Decision algorithm used for retrospective allocation to group

In this study, two groups were constructed retrospectively; there was no control group. The two groups were ‘Planned revision’ and ‘Unplanned revision’ of the hip replacement. Long‐term follow‐up was considered as planned orthopaedic review (including x‐ray) of the hip implant at any time in the period that commenced 5 years after the primary operation to the present day. This group was categorised as ‘Planned revision’. The comparator group were those patients scheduled for revision surgery following an emergency admission or general practitioner (GP) referral due to problems with the hip implant. They were considered as those with no orthopaedic long‐term follow‐up of their hip arthroplasty and/or an ‘Unplanned revision’. The assignment of each participant to one of these two groups was retrospectively completed using a variety of data sources and a decision algorithm (see Figure 1).

### 2.3 Data sources used for decision algorithm

1. Hospital patient information system—inpatient and outpatient attendance data
2. Electronic record of medical notes, including letters to and from GP and other services
3. Radiology archiving systems—imaging of affected hip joint (e.g., the presence of serial images suggests orthopaedic monitoring)

The data gathered from the various sources and knowledge of participating orthopaedic units were incorporated in the decision algorithm to allocate participants to one of two groups—‘Planned revision’ or ‘Unplanned revision’. The ‘12‐month’ cut off from time from referral to revision surgery was used because of current practice at the time of this study. There was commonly a 22–24 weeks wait between GP referral to the orthopaedic service and the first orthopaedic appointment, and then further screening/results and waiting for a pre‐operative appointment, adding another 8–10 weeks. Surgery might take place as soon as 2–3 weeks after the pre‐operative assessment if no complications arose, hence a total of approximately nine months from referral to surgery. The choice of a 12‐month cut off was designed to differentiate between those participants who came to revision surgery without regular orthopaedic assessment (Unplanned revision) and those who were in a regular follow‐up programme or who were being monitored for progression of potentially damaging changes around the THA (Planned revision).

### 2.4 Procedures

The participants in this study were recruited pre‐operatively and completed a set of patient‐reported outcome measures at that time. A self‐reported questionnaire was used to capture use of health resources at 6 months after revision surgery. The patient‐reported outcome measures were repeated 12 months after surgery. Peri‐operative data were collected from medical records.

### 2.5 Primary outcome

The primary clinical outcomes were the difference in patient‐reported measures from the time of surgery to 12 months post‐surgery; the joint specific Oxford Hip Score (OHS; Dawson, Dawson, Fitzpatrick, Carr, & Murray, 1996), the EuroQol EQ‐5D instrument to value quality of life (EuroQol Group, 2019), and the University of Southern California at Los Angeles (UCLA) Activity Score (Naal, Impellizzeri, & Leunig, 2009). For each participant, a change score was constructed by deduction of the pre‐operative score from the 12‐month score.

The OHS is a widely used score consisting of 12 questions about pain and function after total hip replacement (score range from zero, poor outcome, to 48, best outcome). It has been shown to be valid and reliable for use in revision hip surgery (Dawson et al., 2001).

The EuroQol EQ‐5D consists of a self‐report questionnaire Euroqol 5‐dimension Score (EQ‐5D‐5L) which comprises five dimensions (mobility, self‐care, usual activities, pain/discomfort and anxiety/depression) and a visual analogue scale Euroqol Visual Analogue Score (EQ‐VAS) which is a single mark on a scale of 0–100. The EQ‐5D‐5L dimensions were combined to give a single index value with reference to the United Kingdom value set, using the time trade‐off (TTO) method (The EuroQol Group, 2007, p. 21). This produces a score from 1.0, equivalent to full health, through zero (death) to −1.0, a state assumed to be worse than death. The value set for the TTO method was originally constructed from participant ratings of 10 years in several health states in comparison to full health and to death. The TTO method was chosen as it has been shown to be valid for hip revision surgery and use of the United Kingdom value set is recommended by the National Institute for Health and Care Excellence (National Institute for Health and Care Excellence, 2020).

The UCLA activity score consists of 10 statements relating to level of activity and the participant selects the one level that best describes their current state (range from 1 to 10 with a high value representing a more active lifestyle).

### 2.6 Secondary outcome

The secondary outcome measure was the cost of revision hip surgery and the early postoperative period, adopting a health and social care payer perspective for the economic evaluation.

#### 2.6.1 Resource use identification and collection

Data were included for the initial inpatient stay for the revision THA (including operating theatre time, prosthesis and length of stay), subsequent inpatient stays post discharge at any hospital and outpatient visits during the first 12 months post‐revision surgery. The data collected included location, duration and reason for visit.

Volumes for all other resource use were collected using a patient‐reported questionnaire administered at 6 months post‐surgery based on a self‐completed resource use logbook. This included non‐hospital services, medication use, provision of equipment and adaptations made to the home, and use of social services. Non‐hospital services included contacts with GP, practice nurse and district nurse, and physiotherapy and occupational therapy at home or an outpatient clinic. Social services included home care, meal delivery and social worker contact. Participants were asked to include medication, equipment, adaptations, home care and meal provision that were self‐funded.

#### 2.6.2 Valuation of resource use

Volumes of resources and unit cost prices were treated separately. Resources used during the initial hospital stay were valued using unit costs obtained from the hospital finance department. Cost estimates for time spent in theatre and admissions to hospital wards included staff time, overheads, consumables and medications. Prosthesis costs were taken from consortium supply lists or from direct contact with the orthopaedic companies supplying components.

For secondary care visits in the 6‐month post‐operative period, information on the reason for inpatient admission, duration of episode and clinical expert advice was used to derive healthcare resource group codes. Healthcare resource groups and outpatient appointment by clinical speciality were valued using Department of Health Reference Costs (NHS England and NHS Improvement, 2020).

Non‐hospital services and personal social services were valued using unit costs for health and social care (Curtis et al., 2020). Equipment and home adaptations, such as mobility aids, commodes, toilet frames, grab rails and furniture raisers, were financed by health and social care but provided to patients on loan, through occupational therapists and physiotherapists. The useful life of the equipment was assumed to be 2 years and valued as a fraction of equipment cost proportional to the duration of patient use (12 weeks post‐surgery). Unit costs were obtained from the equipment supplier to National Health Service (NHS) Trusts and community services (Medequip Assistive Technologies Ltd.). Items permanently fitted to the property, such as grab rails and stair rails, were valued at full cost. Self‐funded equipment or adaptations were valued as the lowest cost from three online suppliers. Prescribed medication was valued using the British National Formulary (The Royal Pharmaceutical Society, 2020). Resource usage and personal costs incurred due to a pre‐existing medical condition and not directly related to the surgery were excluded. The unit cost estimates can be seen in Table S1.

### 2.7 Statistics and data analysis

This was a pilot study and adopted an exploratory approach to data analysis. Sample size was not predetermined but it was anticipated that 180 participants may be eligible, based on historic data from the orthopaedic unit. Several unanticipated problems led to a reduced number of eligible participants and consequently, there were insufficient primary outcome data for a linear mixed method model. Data are presented with descriptive statistics and tests for the difference between groups, parametric and non‐parametric as indicated by the data distribution (Student's *t*‐test, chi square and Mann–Whitney‐*U* test, significance set at *p* < 0.05; IBM SPSS Statistics 26.0). The economic evaluation of health benefits is presented as a cost‐consequences table in view of the small sample size.

### 2.8 Ethics

Research ethics approval for this study was granted from the NHS Health Research Authority (London—Camden and King's Cross Research Ethics Committee) on 19 April 2016, reference number: 16/LO/0650. All participants gave written informed consent and the study was conducted in accordance with the Declaration of Helsinki.

## 3 RESULTS

**FIGURE 2:**
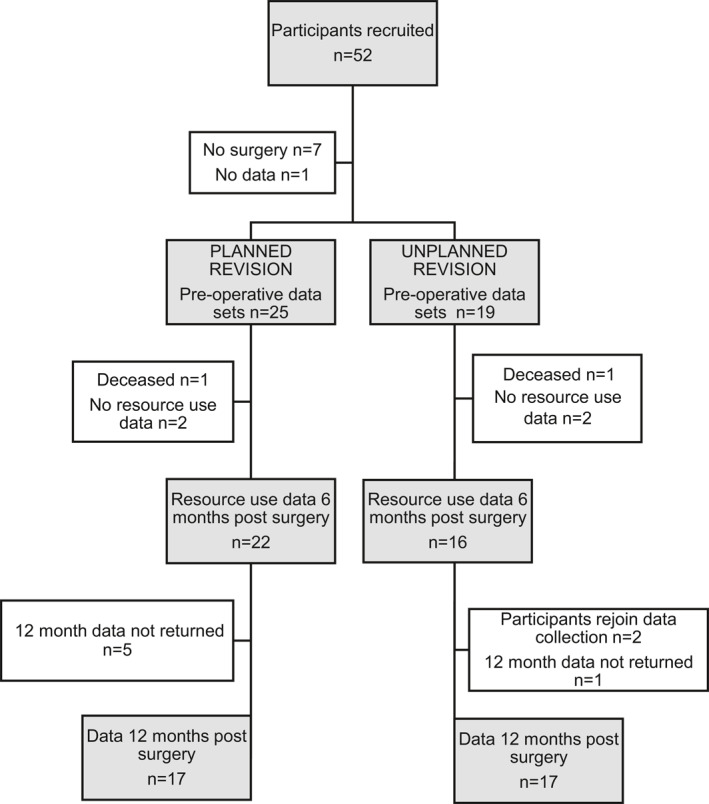
Data collection from participants by group

Between 27 June 2016 and 30 June 2017, 110 patients were screened, 52 gave informed consent and were recruited for the study. The exclusions were as per protocol (Smith et al., 2017) with many patients unwilling or unable to complete a questionnaire 12 months after surgery due to age, comorbidity or cognitive status. The demographics of included participants can be seen in Table 1 and the flow of data collection, by group, in Figure 2. There were no statistically significant differences between groups for any of the baseline patient characteristics.

**TABLE 1:** Demographic details of study participants

Demographic	Planned revision (*n* = 25)	Unplanned revision (*n* = 19)	Statistics, *p*‐value
Age in years, mean, range	73.3 (45.8–86.1)	75.5 (46.5–89.2)	Student's *t* test, 0.49
Sex	10 male	9 male	Chi Squared, 0.63
	15 female	10 female	
Laterality	15 right	11 right	Chi Squared, 0.89
	10 left	8 left	
Charlson comorbidity index, median, range	3.0 (0.0–8.0)	4.0 (1.0–5.0)	Mann–Whitney *U* test, 0.10

Details of the primary reason for revision surgery were extracted from the discharge letter sent to each participant's GP (Table 2). In the Planned revision group, most were undertaken for changes noted on serial X‐rays of the affected hip replacement and in the Unplanned revision group, most were undertaken because of patient‐reported pain.

**TABLE 2:** Primary indication for revision hip arthroplasty

Primary indication for revision	Planned revision (*n* = 25)	Unplanned revision (*n* = 19)
Pain	6 (24%)	11 (58%)
X‐ray changes	13 (52%)	2 (10.5%)
Peri‐prosthetic fracture	0 (0%)	4 (21%)
Dislocation/subluxation	1 (4%)	2 (10.5%)
Adverse reaction to metal debris	5 (20%)	0 (0%)

A summary of the demographic details of the 10 participants with incomplete data sets is presented in Table 3 and shows a male predominance. Table 4 presents a summary of the baseline patient‐reported outcome measures for all participants, showing these to be similar. The primary outcome data were the differences in patient‐reported outcome scores from before to 12‐month after revision surgery (Table 5). There were no statistically significant differences between groups for change scores although the change in EQ‐VAS approached statistical significance.

**TABLE 3:** Demographic details of participants with missing data

Demographic	Planned revision (*n* = 7)	Unplanned revision (*n* = 3)
Age in years, mean, range	72.5 (45.8–81.4)	78.4 (70.2–85.1)
Sex	4 male	2 male
	3 female	1 female
Laterality	4 right	3 right
	3 left	0 left
Charlson comorbidity index, median, range	3.0 (0.0–6.0)	4.0 (3.0–4.0)

**TABLE 4:** Comparison of participants by group: baseline scores and in‐patient data

	Planned revision, participants with complete data (*n* = 17)	Unplanned revision, participants with complete data (*n* = 17)	Participants with incomplete data at 12‐month post‐surgery (*n* = 10)
Age in years, mean	73.3	77.5	70.2
Charlson comorbidity index, median, range	2 (2–8)	3 (1–6)	3 (0–6)
Pre‐operative Oxford hip score, median, range	24.0 (6–41)	23.0 (3–48)	20.5 (11–29)
Pre‐operative EQ‐5D‐5L, median, range	0.449 (−0.346 to 0.735)	0.419 (−0.199 to 0.765)	0.430 (0.051–0.721)
Pre‐operative EQ‐VAS median, range	75 (10–90)	75 (20–95)	72.5 (10–95)
Pre‐operative UCLA score, median, range	4 (2–7)	3 (1–7)	4.5 (3–6)

Abbreviations: EQ‐5D‐5L, Euroqol 5‐dimension Score; EQ‐VAS, Euroqol Visual Analogue Score; UCLA, University of Southern California at Los Angeles activity score.

**TABLE 5:** Change in patient‐reported outcome scores from pre‐ to 12 months post‐revision surgery

Patient reported outcome measure	Planned revision, score changes			Unplanned revision, score changes			Statistics
	Number with complete data = 17			Number with complete data = 17			
	Median	IQR	Range	Median	IQR	Range	Mann–Whitney *U* test, *p*‐value
Φyear Oxford hip score (Basic score 0–48)	13	21	−0.2, +33	11	21	−9, +32	0.50
Φyear EQ‐5D‐5L (Basic score −1.0 to +1.0)	0.254	0.58	−0.51, +1.11	0.223	0.42	−0.5, +0.66	0.92
Φyear EQ‐VAS (Basic score 0–100)	10	18	−10, +68	0	30	−25, +30	0.05
Φyear UCLA (Basic score 1–10)	0	2	−2, +4	0	2	−3, +4	0.51

Abbreviations: EQ‐5D‐5L, Euroqol 5‐dimension Score; EQ‐VAS, Euroqol Visual Analogue Score; IQR, Interquartile range; UCLA, University of Southern California at Los Angeles activity score; Φyear, change over one year.

The major costs associated with the revision hip replacement and hospital stay for all participants are summarised to give an average (median) participant value (Table 6). A notable difference between the two groups occurs in the costs associated with the length of stay in hospital as the unplanned revision group were, on average, in hospital for longer. The median for the Planned revision was 5 days (range 2–22 days) and for Unplanned revision was 11 days (range 3–86 days), which was statistically significant (Mann–Whitney *U* test statistic 333.000, *p* = 0.02). There was no statistically significant difference between the median length of time taken for surgery in the two groups (Planned revision 2.84 h, range 1.14–7.0 h; Unplanned revision 3.04 h, range 1.31–11.0 h; Mann–Whitney *U* test, *p* = 0.84).

**TABLE 6:** Cost Consequences analysis for the in‐patient stay

Cost category	Planned revision, costs (£) *n* = 25			Unplanned revision, costs (£) *n* = 19		
	Median	IQR	Range	Median	IQR	Range
Cost of hospital stay	1135.00	795	454–4994	2497.00	3632	681–19,522
Cost of hip replacement components	1984.62	3135	530–6181	1712.14	1662	774–7361
Cost of time in operating theatre	2726.40	1637	1094–6720	2918.40	1622	1257–10,560
Total costs per patient	6537.47	4322	2892–13,196	8008.56	4771	2713–29,269

Abbreviation: IQR, Interquartile range.

A total of 38 participants completed the resource use questionnaire. Some participants completed the 12‐month questionnaire but not resource use; they found it difficult to concentrate due to comorbidities or carer responsibilities. The data are presented with summary values and show that groups were similar apart from an extended hospital stay for one participant in the Unplanned revision group (Table 7).

**TABLE 7:** Summary of costs from patient‐reported resource use questionnaire in the 6 months post‐surgery

Source of accumulated costs	Resources used per participant (£), median, range	
	Planned revision *n* = 22	Unplanned revision *n* = 16
Use of any community health services since discharge post revision surgery for reasons related to revision hip replacement	78.92 (0.00–831.78)	89.43 (0.00–525.76)
Inpatient in any hospital or rehabilitation unit since discharge for reasons related to hip revision	0.00 (0.00–0.00)	0.00 (0.00–17,290.00)
Outpatient visits at any hospital related to hip revision	0.00 (0–304.00)	0.00 (0.00–222.00)
Use of prescribed medication for reasons related to hip revision	0.00 (0.00–51.48)	0.00 (0.00–111.54)
Use of over‐the‐counter medication for reasons related to hip revision	0.00 (0.00–15.00)	0.00 (0.00–18.00)
Changes made to the home (e.g., grip rails and stair lift) or special equipment provided (e.g., commode, toilet frame, toilet seat and trolley) related to your hip revision	4.31 (0.00–231.46)	4.79 (0.00–229.00)
Requirement for a home care worker (home help) for reasons related to your hip revision	0.00 (0.00–390.00)	0.00 (0.00–0.00)
Use of a home delivery food service for reasons related to your hip revision	0.00 (0.00–50.00)	0.00 (0.00–0.00)
Total patient‐reported resource use, median, range	131.96 (1.75–1291)	251.42 (0–17,378)

## 4 DISCUSSION

This pilot study indicates that changes would be needed in the delivery of any similar future study but offers insight into the effect of long‐term follow‐up services when revision hip arthroplasty is required. The sample was small but demonstrates some differences in patient reported quality of life and peri‐operative costs which favour the use of long‐term follow‐up in this patient population.

The limitations of this work are the size and scope as it was a one‐centre, pilot study and the number of patients recruited were fewer than anticipated due to several factors including patient choice, operational changes, winter pressures and research staff availability. Maintaining the screening of potential participants and recruitment of eligible patients was inconsistent over the 12 months of the study due to staff shortages in research. Of those recruited, a proportion did not proceed to surgery as anticipated, and there was further loss to follow‐up through incomplete data. When compared with those who competed all scores, the only notable difference was a higher proportion of males in this group (Tables 1 and 3). Future studies may be advised to avoid lengthy post‐operative questionnaires for this group of patients and maximise the in‐hospital data collection. In addition, data collection from a large number of hospital units would mitigate for the unforeseen effects that further reduced recruitment in this pilot study. This information is important for future work and contributed to the development of a large, multi‐centre observational study on the effect of long‐term follow‐up services after hip and knee replacement (Czoski‐Murray et al., 2019).

The retrospective allocation of participant to groups (Planned revision vs. Unplanned revision) employed an algorithm which was constructed with reference to clinical knowledge by the authors and orthopaedic colleagues. The variables included time from referral and frequency of appointments in secondary care. It did not differentiate the profession of the referrer, which may provide greater insight into the pre‐revision pathway. It did not attempt to establish if the participant was on a recommended follow‐up pathway, or if the participant had adhered to that pathway. Consequently, some participants may have been incorrectly assigned to a group. However, the allocation was seen to be correct in cases where pathways were known, and the principles incorporated in the algorithm facilitated a consistent approach that could be employed with larger data sets in subsequent work.

The EQ‐VAS requires the patient to indicate on a scale how good or bad they consider their health to be on the day of score completion and provides well‐validated evidence for health quality (EuroQol Group, 2019). Those in the Planned follow‐up group showed a greater improvement over the 12 months after surgery than those without follow‐up, although it did not reach statistical significance (*p* = 0.05). There are many reasons why this score may have shown a difference between groups, such as lower comorbidity scores or a shorter hospital stay. However, one of the possibilities is that the screening, preparation and planned pathway to revision provided by long‐term follow‐up services allows better management of the experience for the patient with a quicker return to health than those without such preparation. This would require further exploration in a larger study.

The cost consequences comparison table (Table 7) shows some benefit to care providers of long‐term follow‐up services, primarily due to the length of stay in hospital which leads to a significant increase in cost if it is prolonged. In this study, the median cost per patient for hospital stay was £1135.00 for the Planned revision group compared to £2497.00 for the Unplanned revision group. This was based on a reference cost for an extra night in hospital of £313.00 (NHS England and NHS Improvement, 2020) and the difference of 6 days in median length of stay. In the United Kingdom, approximately 8000 patients undergo hip revision each year and over 50% of these are for aseptic indications, as in this study (National Joint Registry, 2019). Based on this data, a 6‐day longer stay in hospital for unplanned revision of all aseptic cases would cost an estimated £7.5 million per annum. Although this scenario is unlikely, the potential cost‐savings from providing some long‐term follow‐up to reduce unplanned revision are substantial. Further work is needed to confirm or refute this early finding.

In addition to the costs incurred by an increased length of stay, issues of hospital capacity and bed management are an ongoing, real‐world problem facing hospital management teams and based on this data, would be worsened by the prolonged occupancy of those without follow‐up. A proportion of the unplanned revision group received emergency surgery which may have contributed to the length of stay for reasons such as preparing the patient for surgery or planning for discharge. Emergency revision surgery will always be needed for some patients but there may be others for whom long‐term follow‐up of THA would lead to planned elective revision, thereby minimising the unpredictability of prolonged bed occupancy and its associated problems.

The decision to undertake revision hip arthroplasty is made jointly by the orthopaedic surgeon and the patient. The threshold for a surgeon depends on many factors such as their own experience, the type of components and materials used in the primary arthroplasty, the extent of the damage in the THA and the threat of complete failure of the joint construct. In this study, the primary indication for revision in the Planned revision group was X‐ray change, although some may also have experienced pain (Table 2). This raises a question about the timing of revision surgery as many surgeons are reluctant to operate on an asymptomatic patient and prefer to wait until the patient complains of pain. However, as patients often find it hard to distinguish the source of discomfort until symptoms are severe, their presentation to orthopaedics from primary care may be delayed, either by the patient or by the system. An orthopaedic assessment of the THA provides an opportunity for patient education about symptoms alongside a knowledgeable assessment of the joint construct. In addition, it can provide patients with reassurance which in turn affects mental health and has a social impact, whether that is employment or family responsibility (Arthritis & Musculoskeletal Alliance, 2018).

The delivery of a service that takes place many years after the primary procedure raises questions about individual responsibility and personal attitude—should we be expecting those with a THA to initiate an orthopaedic assessment? There are divided views within the orthopaedic community with some suggesting that the burden of responsibility lies with care providers to keep track of patients and others suggest that it should be entirely patient‐driven with no action until symptoms are such that they re‐present in orthopaedics (Rose, Dures, & Smith, 2020). A model of service delivery which can offer rapid access for those with concerns about a joint replacement, in addition to continuous surveillance for high‐risk patients, may be more acceptable to service users than no service or too‐frequent reviews, and may incur fewer problems with patient non‐attendance. For instance, based on current registry reports, an opt‐in arthroplasty review 10 years after the primary surgery could provide reassurance and assessment at the start of the second decade of the joint replacement. This requires further exploration as many in the orthopaedic community consider that the current guidelines of review at 1, 7 and 10 years, and 3‐yearly thereafter are unsustainable but that a modified service should be offered to the THA patient population (British Hip Society et al., 2019; Cassidy et al, 2019; Rose et al., 2020).

## 5 CONCLUSION

This pilot study has shown that modifications to the methods would be needed in any further study. The results suggest that there may be some financial benefit from providing patients with a follow‐up service in the long‐term after THA but the effect on patients is unclear from this small sample. A larger study is needed to explore these findings and to discuss the impact on current recommended guidelines.

## CONFLICT OF INTEREST

The authors report no conflicts of interest in this work.

## AUTHOR CONTRIBUTIONS

Shea Palmer, Jane Powell, Erik Lenguerrand and Lindsay K. Smith contributed to the design and implementation of the research, to the analysis of the results and to the writing of the manuscript. Emma Turner contributed to collection and analysis of health economic data, and to the writing of the manuscript.

## Supporting information

Supplementary Material
