# Supplementary material for: Pilot study: Is a long‐term follow‐up service beneficial for patients undergoing revision hip replacement surgery?
Source: Musculoskeletal Care. 2020 Oct 21;19(3):259–68. doi: 10.1002/msc.1521 (PMC8518955; doi:10.1002/msc.1521)
Supplement: Supplementary file 1 — Supplementary Material [file MSC-19-259-s001.docx]

# Appendix I. Costs attributed to participants

All costs in GBP (British pound sterling).

Key: GP=general practitioner; PSSRU=Personal Social Services Research Unit^17^

| **Equipment Costings** | |  |  |  |  |  |  |
| --- | --- | --- | --- | --- | --- | --- | --- |
| Medequip online NHS catalogue 08/18) | |  |  |  |  |  |  |
|  |  |  |  |  |  |  |  |
| **Description** | **Product Code** | **Cost per unit** | **2-year write down cost** | **3-month use write down cost** |  |  |  |
| Free standing toilet frame | TOI723 | 14.00 | 7.00 | 1.75 |  |  |  |
| Raised toilet seat (RTS) | TOI701 | 5.70 | 2.85 | 0.71 |  |  |  |
| Commode | TOI729 | 19.50 | 9.75 | 2.44 |  |  |  |
| Chair raises | SEA640 | 13.99 | 7.00 | 1.75 |  |  |  |
| Sofa raise | 2x(SEA701+SEA711) | 20.84 | 10.42 | 2.61 |  |  |  |
| Bed raise | BED407 | 15.74 | 7.87 | 1.97 |  |  |  |
| Bed lever multipurpose | BED485 | 25.00 | 12.50 | 3.13 |  |  |  |
| Bed lever slatted | BED908 | 34.99 | 17.50 | 4.37 |  |  |  |
| Bed lever divan | BED228B | 34.99 | 17.50 | 4.37 |  |  |  |
| Perching stool | KIT400PU | 22.80 | 11.40 | 2.85 |  |  |  |
| Trolley | KIT412 | 21.50 | 10.75 | 2.69 |  |  |  |
| Gutter frame | MOB551 | 60.52 | 30.26 | 7.57 |  |  |  |
| 3-wheeled walker | MOB565 | 30.00 | 15.00 | 3.75 |  |  |  |
| Wheeled zimmer frame | MOB631 | 14.50 | 7.25 | 1.81 |  |  |  |
| Pair crutches | MOB704 | 8.09 | 4.05 | 1.01 |  |  |  |
| Metal stick | MOB590 | 2.30 | 1.15 | 0.29 |  |  |  |
| Shower seat | BAT210 | 12.95 | 6.48 | 1.62 |  |  |  |
| Back sponge | not in catalogue |  |  |  |  |  |  |
| Shoehorn | BED279 | 0.70 | 0.35 | 0.09 |  |  |  |
| Grab rail plastic shower | BAT141F | 2.69 | 1.35 | 0.34 |  |  |  |
| Grab rail outside metal | BAT191F | 1.43 | 0.72 | 0.18 |  |  |  |
| Orthopaedic chair | SEA630 | 68.00 | 34.00 | 8.50 |  |  |  |
|  |  |  |  |  |  |  |  |
| **Other not self-funded: not in Medequip catalogue** | |  |  |  |  |  |  |
| Electric chair (riser recliner) | Livewell fabric | 339.00 |  |  |  |  |  |
| Stair rail 3m NHS | Care and Repair | **40.00** |  |  |  |  |  |
|  |  |  |  |  |  |  |  |
| **Post Discharge Hospital and Community Based Health Services** | | | |  |  |  |  |
|  |  |  |  |  |  |  |  |
| **Non-inpatient Services (A)** |  | **Cost GBP** |  |  |  |  |  |
| GP surgery visit |  | 37.00 | base cost per patient contact of GP with qualifications inc direct care staff costs (GBP 242 per hour) , lasting 9.22min (PSSRU 2017:10.3b) | | | | |
| GP home visit |  | 94.38 | base cost per out of surgery visit of GP with qualifications inc direct care staff costs (GBP 242 per hour) (PSSRU 2017:10.3b), lasting 23.4min (last quoted in PSSRU 2015: 10.8a) | | | | |
| Phone GP |  | 28.40 | base cost per telephone consultation with GP with qualifications including direct care staff costs (GBP 242 per hour) (PSSRU 2017: 10.3b), lasting 7.1 mins (last quoted in PSSRU 2015: 10.8a) | | | | |
| Practice nurse surgery visit |  | 10.85 | base cost per practice nurse contact using base cost 42.00/hour of GP nurse time with qualifications (PSSRU 2017: 10.2), lasting 15.5 mins (last quoted in PSSRU 2015: 10.6) | | | | |
| Repeat Prescription |  | 12.00 | base on assumption of 3 mins of GP time using base cost of one minute of GP time (GBP 4.0) with qualifications including direct care staff costs GBP 242 (PSSRU 2017: 10.3b) (as per Apex) | | | | |
| District Nurse |  | 22.00 | based on assumption of 15 mins contact time plus 15mins travel time using base cost 44.00/hour band 6 community nursing time (PSSRU 2017: 10.1) | | | | |
| OT home visit |  | 43.00 | based on 60 mins contact time including travel using base cost 43.00/hour of band 6 OT contact (PSSRU 2017:9) | | | | |
| OT in GP surgery or clinic |  | 22.50 | based on 30 mins contact time using base cost 45.00/hour of hospital based band 6 OT contact (PSSRU 2017: 10.6) | | | | |
| Physio in hospital outpatients |  | 22.50 | based on 30 mins contact time using base cost 45.00/hour of hospital based band 6 PT contact (PSSRU 2017: 10.6) | | | | |
| Physio home visit |  | 43.00 | based on 60 mins contact time including travel using base cost 43.00/hour of band 6 PT contact (PSSRU 2017: 9) | | | | |
| Home care |  | 26.00 | based on one hour of face to face weekday contact per day for independent sector home care provided for social services (PSSRU 2017:11.6) | | | | |
|  |  |  |  |  |  |  |  |
| **Medication** |  |  |  |  |  |  |  |
|  |  |  |  |  |  |  |  |
| **Tablets** | **Recommended dose (A)** | **Tablets** | **Daily dose (no.)** | **Weekly dose, strength (B)** | **Number in pack (B)** | **Unit cost GBP (C)** | **Cost per week GBP** |
| Paracetamol | 500mg -1g (max four times a day) | 8 x 500mg | 56 | 500mg | 100 | 0.87 | 0.49 |
| Codeine Phosphate | 30-60mg every 4 hours (max 240mg/day) | 8 x 30mg | 56 | 30mg | 28 | 0.80 | 1.60 |
| Prednisolone 5mg | 5mg daily | 1 x 5mg | 7 | 5mg | 28 | 0.60 | 0.15 |
| Indametacin | 50mg-100mg daily divided doses | 2 x 50mg | 14 | 50mg | 28 | 1.21 | 0.61 |
| Tramadol | 50-100mg every 4-6 hours max 400mg/day | 8 x 50mg | 56 | 50mg | 60 | 4.60 | 4.29 |
| Amitriptyline | 25mg-75mg daily | 1 x 50mg | 7 | 50mg | 28 | 2.50 | 0.63 |
| Dihydrocodeine/  paracetamol (codydramol) | 10/500mg to 20/1000mg every 4-6 hours (max 80/4000mg per day) | 8 x 10/500mg | 56 | 10/500mg | 30 | 0.62 | 1.16 |
| Naproxen | 250mg every 6-8 hours | 4 x 250mg | 28 | 250mg | 28 | 0.88 | 0.88 |
| Nurofen | 200-400mg every 4-6 hours | 8 x 200mg | 56 | 200mg | 16 | 2.30 | 8.05 |
|  |  |  |  |  |  |  |  |
| **Other** |  |  |  |  |  |  |  |
| Transdermal BuTrans Patch | 20 micrograms/hour patch for one week | N/A | 1 patch | 20mg | 4 x patches | 57.46 | 14.37 |
| Oramorph | 5mg every 4 hours (10mg/5ml) | 20mg (10ml) | 140mg (70ml) | 10mg/5ml | 100ml | 1.89 | 1.32 |
|  |  |  |  |  |  |  |  |
|  | **Key:** |  |  |  |  |  |  |
|  |  | (A) Dose for moderate musculoskeletal pain. Source BNF online htpps://www.medicinescomplete.com accessed 5/10/18 | | | | | |
|  |  | (B) Unit (strength and number per pack) per BNF htpps://www.medicinescomplete.com accessed 5/10/18 | | | | | |
|  |  | (C) NHS Indicative Price per unit BNF htpps://www.medicinescomplete.com accessed 5/10/18 | | | | |  |
